# Galactodendritic Phthalocyanine Targets Carbohydrate-Binding Proteins Enhancing Photodynamic Therapy

**DOI:** 10.1371/journal.pone.0095529

**Published:** 2014-04-24

**Authors:** Patrícia M. R. Pereira, Sandrina Silva, José A. S. Cavaleiro, Carlos A. F. Ribeiro, João P. C. Tomé, Rosa Fernandes

**Affiliations:** 1 QOPNA and Department of Chemistry, University of Aveiro, Aveiro, Portugal; 2 Laboratory of Pharmacology and Experimental Therapeutics, Institute for Biomedical Imaging and Life Sciences (IBILI), Faculty of Medicine, University of Coimbra, Azinhaga de Santa Comba, Coimbra, Portugal; 3 Department of Organic Chemistry, Ghent University, Gent, Belgium; 4 Center of Investigation in Environment, Genetics and Oncobiology, Coimbra, Portugal; 5 Center of Ophthalmology and Vision Sciences, IBILI, Faculty of Medicine, University of Coimbra, Coimbra, Portugal; MGH, MMS, United States of America

## Abstract

Photosensitizers (PSs) are of crucial importance in the effectiveness of photodynamic therapy (PDT) for cancer. Due to their high reactive oxygen species production and strong absorption in the wavelength range between 650 and 850 nm, where tissue light penetration is rather high, phthalocyanines (Pcs) have been studied as PSs of excellence. In this work, we report the evaluation of a phthalocyanine surrounded by a carbohydrate shell of sixteen galactose units distributed in a dendritic manner (PcGal_16_) as a new and efficient third generation PSs for PDT against two bladder cancer cell lines, HT-1376 and UM-UC-3. Here, we define the role of galacto-dendritic units in promoting the uptake of a Pc through interaction with GLUT1 and galectin-1. The photoactivation of PcGal_16_ induces cell death by generating oxidative stress. Although PDT with PcGal_16_ induces an increase on the activity of antioxidant enzymes immediately after PDT, bladder cancer cells are unable to recover from the PDT-induced damage effects for at least 72 h after treatment. PcGal_16_ co-localization with galectin-1 and GLUT1 and/or generation of oxidative stress after PcGal_16_ photoactivation induces changes in the levels of these proteins. Knockdown of galectin-1 and GLUT1, via small interfering RNA (siRNA), in bladder cancer cells decreases intracellular uptake and phototoxicity of PcGal_16_. The results reported herein show PcGal_16_ as a promising therapeutic agent for the treatment of bladder cancer, which is the fifth most common type of cancer with the highest rate of recurrence of any cancer.

## Introduction

Conventional photodynamic therapy (PDT) combines a non-toxic photosensitizer (PS), light irradiation at a specific wavelength and tissue molecular oxygen to produce cytotoxic reactive oxygen species (ROS) [1], [2]. The molecular mechanisms underlying PDT are not clearly understood. However, it has been described that the generation of ROS will trigger signalling pathways that ultimately destroy the targeted tissue. Cell death in PDT may occur by apoptotic and by non-apoptotic mechanisms (*e.g.* necrosis), or even by a combination of the two mechanisms [2]. Additionally, studies suggest that cell death pathway induced after PDT depends on the PS and its intracellular localization, the PDT dose and the cell metabolic potential (*e.g.* its intrinsic antioxidant capacity) [2]. To enhance the specific deliver/target of PSs in cancer cells, third generation PSs have been synthesized, by conjugating them with biochemical motifs [3]–[5]. Among new third generation PSs, the advances in the past years concerning glycobiology have spurred the development of carbohydrate-based molecules for cancer treatment by PDT [3], [4], [6]–[14].

Carbohydrates have a strong potential as PS-delivery systems, because they are biocompatible molecules with a rapid cellular uptake and specific recognition by lectin proteins, which play an important role in several biochemical signalling pathways implicated in cancer metastasis, cell growth and inflammation [15], [16]. The exact interaction mechanism of PS-carbohydrate conjugates with cancer cells is still unknown. However, it is expected that the specific (non-covalent) binding of carbohydrates with lectins [16], promotes the accumulation of the glyco-conjugate inside cells by the endocytic pathway. In addition, the expression of certain carbohydrate-binding lectins (*e.g.* galectins) is higher in cancer cells than in non-tumoral cells [17].

Among carbohydrates, the biocompatibility of galactose molecules and their specific recognition by galectins overexpressed in cancer cells (*e.g.* galectin-1 and galectin-3 [18]) have led to the development of galacto-conjugated PSs. Besides galectins, galactose carbohydrates can bind to GLUT1 (a well-known glucose transporter [19]–[21]). The steriospecificity of GLUT1 (recognizing both D-glucose and D-galactose) has been reported [19]–[21]. Galactose is a C4 epimer of glucose that can bind the glucose-binding site of GLUT1. There is strong evidence in literature that conjugation of carbohydrates (monosaccharides such as glucose and galactose, disaccharides such as lactose) with porphyrinoids [6], [8], [9], [22]–[30] can improve the accumulation of PSs in cancer cells and, consequently, their photoactivity. Furthermore, it has been reported a marked contrast in terms of adsorption on the cells between galactose and glucose conjugated PSs. The former presented a selective uptake by rat hepatoma RLC-116 cells [29].

Recently, the emerging role of dendrimers (with well-defined nano-scaled structures) in biological systems has highlighted their potential benefits for the preparation of new anticancer drugs [31]–[33]. Regarding dendritic units of specific carbohydrates, it is well-known their multivalent interactions with lectins, promoting a synergistic increase in binding affinity [31]. The photodynamic efficiency of porphyrins conjugated with glycodendrimers has been reported in the literature [12], [34]–[37]. However, the *in vitro* PDT studies with the corresponding phthalocyanines (Pcs) are scarce.

Recently, we have reported the synthesis of a new Pc decorated with sixteen molecules of galactose (in a dendritic manner, PcGal_16_, Figure S1) [34]. PcGal_16_ demonstrated strong absorbance in the red spectral region (600–800 nm), fluorescence emission bands at 734 and 805 nm, solubility in a phosphate buffered saline (PBS) solution and interaction with human serum albumin [34]. Additionally, PcGal_16_ demonstrated photostability and ability to generate ROS after photoactivation. The present study was undertaken to validate the *in vitro* photodynamic efficacy of this PcGal_16_ from the standpoint of its uptake by bladder cancer cells (HT-1376 and UM-UC-3, derived from transitional cell carcinoma) to interaction with carbohydrate-binding proteins; induction of phototoxicity, ROS production and activity of antioxidant enzymes after PDT. Our findings show that PcGal_16_ has a strong photodynamic efficiency in an *in vitro* system of bladder cancer.

## Materials and Methods

### Synthesis of galacto-dendrimer phthalocyanine (PcGal_16_)

PcGal_16_ was synthesized as previously described [34]. Zinc 1,2,3,4,8,9,10,11,15,16,17,18,22,23,24,25-hexadeca-fluoro-phthalocyaninato zinc(II) (PcF_16_) was obtained from Sigma. Stock solutions of PSs were prepared at a concentration of 2 mM in dimethyl sulfoxide (DMSO; Sigma-Aldrich, St Louis, MO, USA). Working solutions of PcGal_16_ 0.5–9 µM were freshly prepared in sterile phosphate-buffered saline (PBS) keeping the concentration of DMSO always lower than 0.45% (v/v).

### Cells culture and treatments

Human bladder cancer cell lines UM-UC-3 and HT-1376 derived from high-grade transitional cell carcinoma (from the American Type Culture Collection, ATCC, Manassas, VA, USA) were cultured in Eagle's Minimum Essential Medium (EMEM; ATCC) supplemented with 10% (v/v) of fetal bovine serum (Life Technologies, Carlsbad, CA, USA), 100 U/mL penicillin, 100 µg/mL streptomycin and 0.25 µg/mL amphotericin B (Sigma).

UM-UC-3 and HT-1376 cells were seeded at a density of 3×10^4^ and 4×10^5^ cells/well in 96- and 6-well culture plates (Orange Scientific, Braine-l'Alleud, Belgium), respectively. Twenty-four hours after plating, cells were incubated with the desired concentrations of PSs in the dark for the indicated period of time.

Photodynamic irradiation was carried out in fresh culture medium, devoid of PS, covering UM-UC-3 and HT-1376 cell monolayers and exposing them to red light (620–750 nm) delivered by an illumination system (LC-122 LumaCare, London). The light was delivered for 10 min or 40 min at a fluence rate of 2.5 mW/cm^2^ or 10 mW/cm^2^, as measured with an energy meter (Coherent FieldMaxII-Top) combined with a Coherent PowerSens PS19Q energy sensor [34]. Sham-irradiated cells, used as controls, consisted in cells kept in the dark for the same durations and under the same environmental conditions as the irradiated cells. In all treatments, triplicate wells were established under each experimental condition, and each experiment was repeated at least three times.

### Cellular uptake of PcGal_16_

After incubation with PcGal_16_ in the dark, UM-UC-3 and HT-1376 cells were immediately washed with PBS buffer and lysed in 1% m/v sodium dodecyl sulfate (SDS; Sigma) in PBS buffer at pH 7.0. PcGal_16_ intracellular concentration was determined by spectrofluorimetry using an IVIS Lumina XR equipment (Caliper Life Sciences, Hopkinton MA) with excitation and emission wavelengths set at 675 nm and Cy 5.5 (695–770 nm), respectively, and the results were normalized for protein concentration (determined by bicinchoninic acid reagent; Pierce, Rockford, IL, USA).

For microscopic evaluation, UM-UC-3 and HT-1376 bladder cancer cells were grown for 24 h on glass coverslips coated with poly-L-lysine (Sigma). The cells were incubated with 5 µM PcGal_16_ for 2 h, at 37°C. After incubation, cells were fixed with 4% paraformaldehyde (PFA; Merck, Darmstadt, Germany) for 10 min at room temperature. The samples were then rinsed in PBS, and mounted in VectaSHIELD mounting medium containing 4′,6-diamidino-2-phenylindole (DAPI; Vector Laboratories, CA, Burlingame) for visualization under a confocal microscope (LSM 510, Carl Zeiss, Gottingen, Germany). For detection of PcGal_16_, the specimen was excited at 633 nm and its emitted light was collected between 653–750 nm. For DAPI detection, specimen was excited at 405 nm and its emitted light was collected between 430–500 nm.

### Cell metabolic activity and membrane integrity

#### Trypan Blue dye exclusion

Cell membrane integrity after PcGal_16_ incubation in the dark, irradiation, or both was determined by the trypan blue dye (Biowhittaker, Walkersville, MD, USA) exclusion test 24, 48 and 72 h after each treatment. Cells with intact membrane were counted on a Neubauer chamber after trypsinization and the cell viability of treated cells was normalized to that of the untreated cells.

#### MTT assay

Cell metabolic activity after PcGal_16_ incubation in the dark, irradiation, or both was determined 24, 48 and 72 h after treatments by measuring the ability of bladder cancer cells to reduce 3-[4,5-dimethylthiazol-2-yl]-2,5-diphenyl-tetrazolium bromide (MTT, Sigma), to a colored formazan using a microplate reader (Synergy HT, Biotek, Winooski, VT, USA). The data were expressed in percentage of control (*i.e.* optical density of formazan from cells not exposed to PcGal_16_).

IC_50_ values (*i.e.* concentration of PcGal_16_ required to reduce cell viability by 50% as compared to the control cells) were calculated using non-linear regression analysis to fit dose-response curves in GraphPad Prism 5.0 software (La Jolla, CA, USA).

### Detection of intracellular Reactive Oxygen Species (ROS) generation

Immediately after irradiation or sham-irradiation, cancer cells were washed twice with PBS and incubated with either 2 or 5 µM 2′,7′-dichlorodihydrofluorescein diacetate (H_2_DCFDA; Invitrogen Life Technologies, Carlsbad, CA, USA) for an additional 1 h period, at 37°C, protected from light. After incubation, cells were washed with PBS and lysed in 1% (m/v) SDS solution in PBS (pH 7.0). DCF fluorescence was determined using a microtiter plate reader (Synergy HT) with the excitation and emission filters set at 485/20 nm and 528/20 nm, respectively. Protein concentration was determined using the Pierce BCA Protein Assay Kit.

The ROS levels were also qualitatively evaluated by fluorescence microscopy. After PDT treatments, UM-UC-3 and HT-1376 human bladder cancer cells grown on coverslips were incubated with 5 µM of H_2_DCFDA in PBS buffer (in dark conditions). After washing steps and fixation in 4% (m/v) PFA, coverslips were mounted using VectaSHIELD mounting medium and the slides were visualized under a confocal microscope (LSM 710, Carl Zeiss).

### Redox quenching studies

Immediately after PcGal_16_ uptake, photodynamic treatment was performed with cell monolayers covered with culture medium containing 50 nM of redox quenchers sodium azide, L-histidine and L-cysteine obtained from Sigma. The effect of quenchers on cell viability was evaluated 24 h after PDT by the MTT viability assay.

### TUNEL assay

Cell death was detected by terminal deoxynucleotidyltransferasedUTP nick end-labeling (TUNEL) assay, using the DeadEnd Fluorometric TUNEL System (Promega, Madison, WI, USA), according to the manufacturer's instructions. Briefly, 24 and 72 h after PDT treatment, bladder cancer cells were fixed in 4% (m/v) PFA and permeabilized with 0.2% v/v Triton X-100 in PBS solution. Cells were stained with TdT reaction cocktail for 60 min at 37°C. The nuclei were stained with DAPI and the cells were analyzed under a fluorescence microscope (Leica DFC350 FX, Leica Microsystems, Bannockburn, IL, USA). Tunel-positive DAPI-stained cells were counted in 10 randomly selected fields from three independent experiments. Percentage of dead cells was expressed as ratio of TUNEL-positive cell numbers to DAPI-stained cell numbers.

### Antioxidant enzyme activities

Cell homogenates were obtained immediately after PDT and centrifuged at 10,000 g for 10 min at 4°C. The supernatants were used for measurements of glutathione peroxidase (GPox), glutathione reductase (GR), glutathione S-transferase (GST), superoxide dismutase (SOD) and catalase (CAT) activities in 96-well plates using a Biotek Synergy HT spectrophotometer (Biotek). The activity was expressed as nmol of substrate oxidized per minute per mg of protein (mU/mg).

GPox activity was determined at 30°C, measuring the NADPH (Merck) oxidation at 340 nm. Supernatants were mixed with 1 mM of glutathione-reduced form (GSH; Sigma), 0.5 U/mL GR (Sigma), 0.18 mM NaDPH, 1 mM EDTA (Sigma) and 0.7 mM *tert*-butyl hydroperoxide (t-BOOH; Sigma) in 50 mM imidazole (Sigma) at pH 7.4. The activity was calculated using the NADPH extinction coefficient of 0.62 m^2^/mmoL.

GR activity in cell supernatants was determined at 30°C by measuring the rate of NADPH oxidation at 340 nm in the presence of 3 mM glutathione-oxidised form (Sigma), 0.12 mM NADPH, and 2.5 mM EDTA, in 50 mM Hepes (pH 7.4). The activity was calculated using the NADPH extinction coefficient of 0.62 m^2^/mmoL.

GST activity was determined at 30°C by monitoring the formation of GSH conjugate with 1-chloro-2,4-dinitrobenzene (CDNB; Sigma) at 340 nm in the presence of 1 mM GSH and 1 mM CDNB in 50 mM Hepes (pH 7.4). The activity was calculated using the conjugate extinction coefficient of 0.96 m^2^/mmoL.

SOD activity was determined at 25°C measuring the cytochrome c (Merck) reduction at 550 nm. The supernatants were mixed with 40 µM cytochrome c solution (0.05 M potassium phosphate, 0.5 mM EDTA, pH 7.8) containing 80 µM xanthine (Merck). To initiate the reaction, 2 U/mL xanthine oxidase (Merck) was added. The increase in cytochrome c absorbance at 550 nm was recorded. SOD activity was calculated considering that one unit of SOD activity represents the inhibition of 50% in the rate of increase in absorbance at 550 nm when compared with control (sample without SOD under the conditions of the assay).

CAT activity was determined at 25°C by monitoring the rate of hydrogen peroxide (0.04% w/w) decomposition in 0.05 M potassium phosphate, pH 7.0. One unit of catalase activity was defined by the enzyme quantity that produced an absorbance reduction of 0.43 per minute at 240 nm in this system.

### Transfection assays

Galectin-1 or GLUT1 was depleted in human bladder cancer cells using a pool of three target-specific 20–25 nt siRNA (Santa Cruz Biotechnology, Inc., Santa Cruz, CA, USA). UM-UC-3 and HT-1376 bladder cancer cells were transfected in 6- or 96-well culture plates, at 60–80% confluence, with galectin-1 and GLUT1, respectively. Cells were also transfected with a scrambled siRNA in parallel as controls.

For each transfection, cells were treated for 5 h with 2.4 µM of siRNA in transfection medium (Santa Cruz) containing 0.5 µL/cm^2^ of transfection reagent (Santa Cruz). After incubation, complete media was added and the cells were incubated for 24 or 48 h. Galectin-1 or GLUT1 downregulation was evaluated 24 h or 48 h post-transfection by Western blotting. The uptake and PDT experiments were performed 24 h or 48 h post-transfection with GLUT1 hsiRNA or galectin-1 hsiRNA, respectively.

### Western blot

After PDT treatment, UM-UC-3 and HT 1376 cells were washed twice with ice-cold PBS and harvested in RIPA buffer (150 mM NaCl, 50 mM Tris-HCl, pH 7.5, 5 mM ethylene glycol tetraacetic acid (EGTA), 1% Triton X-100, 0.5% sodium deoxycholate (DOC), 0.1% SDS, 2 mM phenylmethanesulfonyl (PMSF), 2 mM iodoacetamide (IAD),) and 1× protease inhibitor cocktail (Roche, Indianapolis, IN, USA)). After centrifugation at 16,000 g for 10 min at 4°C, supernatants were used for protein quantification using the Pierce BCA Protein Assay Kit, followed by denaturation of the sample with Laemmli buffer. For the Western Blotting analysis, 60 µg proteins were loaded per lane on sodium dodecyl sulphate-polyacrilamide gels (SDS-PAGE). Following electrophoresis and transfer to PVDF membranes (Bio-Rad, Hercules, CA, USA), the blots were incubated in 5% (m/v) nonfat milk in TBS-T (20 mM Tris, 150 mM NaCl, Tween 0.2%, pH 7.6) and probed with rabbit anti-galectin-1 1∶1,000 (Abcam, Cambridge, UK), rabbit anti-GLUT1 1∶1,000 (Chemicon, Boston, MA, USA) and mouse anti β-actin 1∶20,000 (Sigma) antibodies. After washing, the membranes were probed with secondary anti-rabbit or anti-mouse IgG-HRP-linked antibodies (1∶10,000; Bio-Rad). Immunoreactive bands were detected by enhanced chemiluminiscence (ECL) substrate using an imaging system (VersaDoc 4000 MP, Bio-Rad) followed by densitometric analysis.

### Immunofluorescence

UM-UC-3 and HT-1376 human bladder cancer cells were grown on coverslips as previously described [20], [21]. After treatment, cells were washed with PBS and fixed in 4% PFA. Cells were then permeabilized with 1% Triton X-100 in PBS (pH 7.4) and blocked with 5% bovine serum albumin in PBS buffer, before incubation with primary antibodies rabbit anti-galectin-1 1∶100 (Abcam) and rabbit anti-GLUT1 1∶250 (Chemicon). The cells were then rinsed with PBS buffer and incubated with DAPI and secondary fluorescent antibodies. After washing, samples were imaged using a confocal microscope (LSM 710, Carl Zeiss).

### Statistical analysis

The results are presented as mean ± standard deviation (S.D.) with n indicating the number of experiments. Statistical significance among two conditions was assessed using the nonparametric Mann-Whitney test. Statistical significance among three conditions was assessed by the nonparametric Kruskal-Wallis test. Statistical significance among several conditions was assessed with the Friedman test. P-value was considered at the 5% level of significance to deduce inference of the significance of the data. All graphs and statistics were prepared using the GraphPad Prism 5.0 software.

## Results

### PcGal_16_ accumulates in cancer cells and is non-toxic in darkness

**Figure 1:**
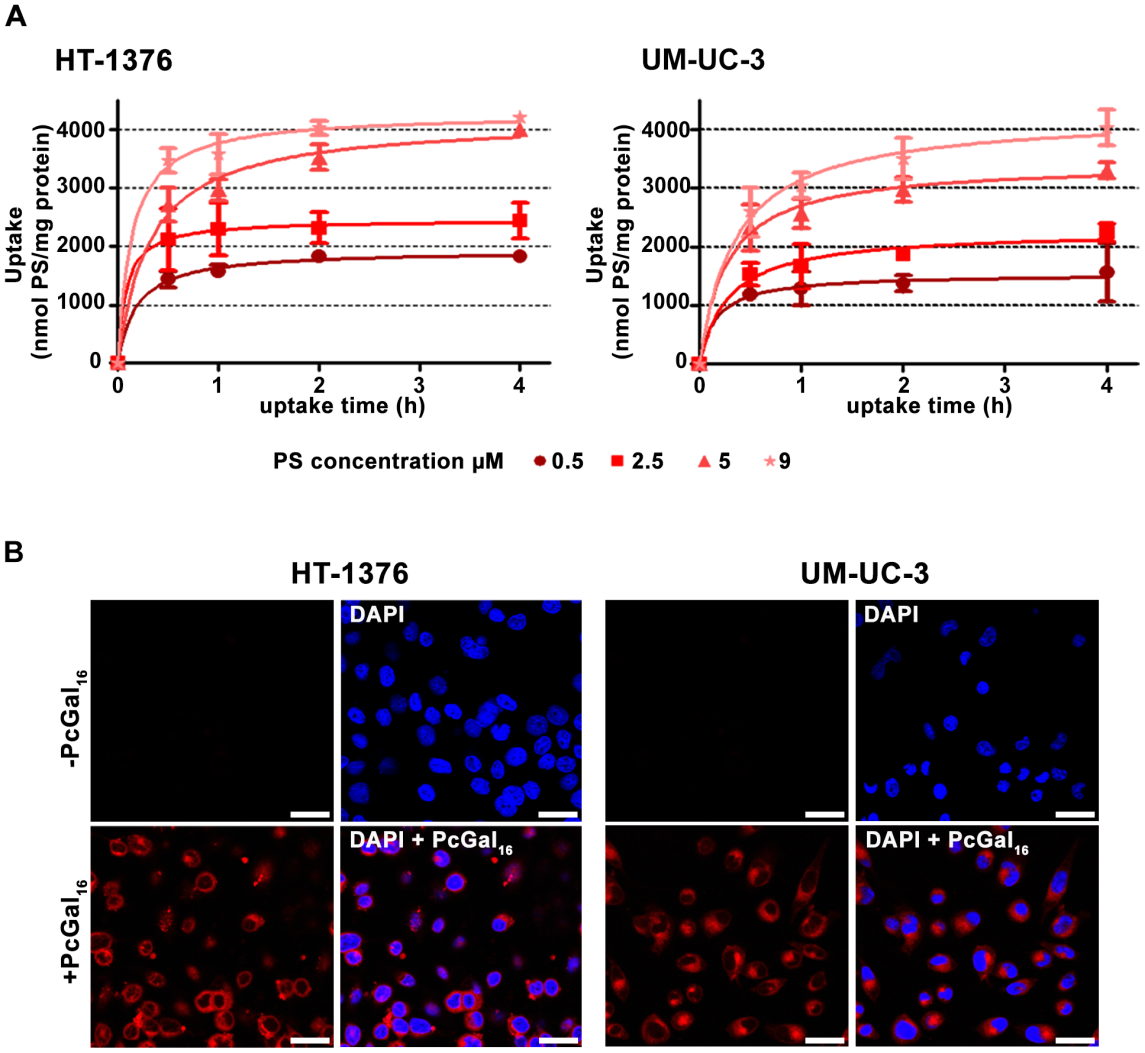
PcGal_16_ accumulates in UM-UC-3 and HT-1376 human bladder cancer cells. Intracellular uptake of PcGal_16_ by HT-1376 and UM-UC-3 bladder cancer cells (panel A). The concentration of PcGal_16_ was determined by fluorescence spectroscopy and the results were normalized to protein quantity. Data are the mean ± S.D. of at least three independent experiments performed in triplicates. Representative fluorescence images (panel B) of bladder cancer cells incubated with PcGal_16_ (red) in darkness and cell nucleus stained with DAPI (blue). *Scale bars* 20 µm.

To study the cellular uptake of PcGal_16_, HT-1376 and UM-UC-3 bladder cancer cells have been incubated with increasing concentrations (0.5, 2.5, 5 and 9 µM) of PcGal_16_ in PBS for up to 4 h. PcGal_16_ intracellular accumulation was determined by quantitative spectrofluorimetry and fluorescence microscopy. As shown in Figure 1A, the uptake of PcGal_16_ was both concentration- and time- dependent, reaching a plateau in less than 2 h. Addition of 5 µM PcGal_16_ to HT-1376 and UM-UC-3 cells resulted in an intracellular concentration of 3531±125.9 and 2973±119.1 nmol PcGal_16_ per mg of protein, respectively, after 2 h of incubation (Figure 1A). This spectrofluorimetric data was confirmed by confocal microscopy showing that cells treated with PcGal_16_ exhibit strong fluorescence, with occasional bright spots in the perinuclear region (Figure 1B). PcF_16_, the non-conjugated Pc (Figure S1), was used as control. No significant intracellular accumulation was observed when the cells were incubated with 0.5–9 µM PcF_16_ (data not shown), showing that the uptake of the PcGal_16_ by cancer cells is enhanced relatively to unconjugated PcF_16_. After confirmation of PcGal_16_ uptake by bladder cancer cells, its cytotoxic effect in darkness was assessed by the MTT colorimetric assay (Figure S2). No dark toxicity was observed in untreated cells (up to 4 h) in the presence of 0.45% or less DMSO in the incubation medium. Moreover, PcGal_16_ showed no significant cytotoxicity at concentrations up to 9 µM up to 72 h after treatment (Figure S2).

### PcGal_16_ induces cytotoxicity after photodynamic activation

**Figure 2:**
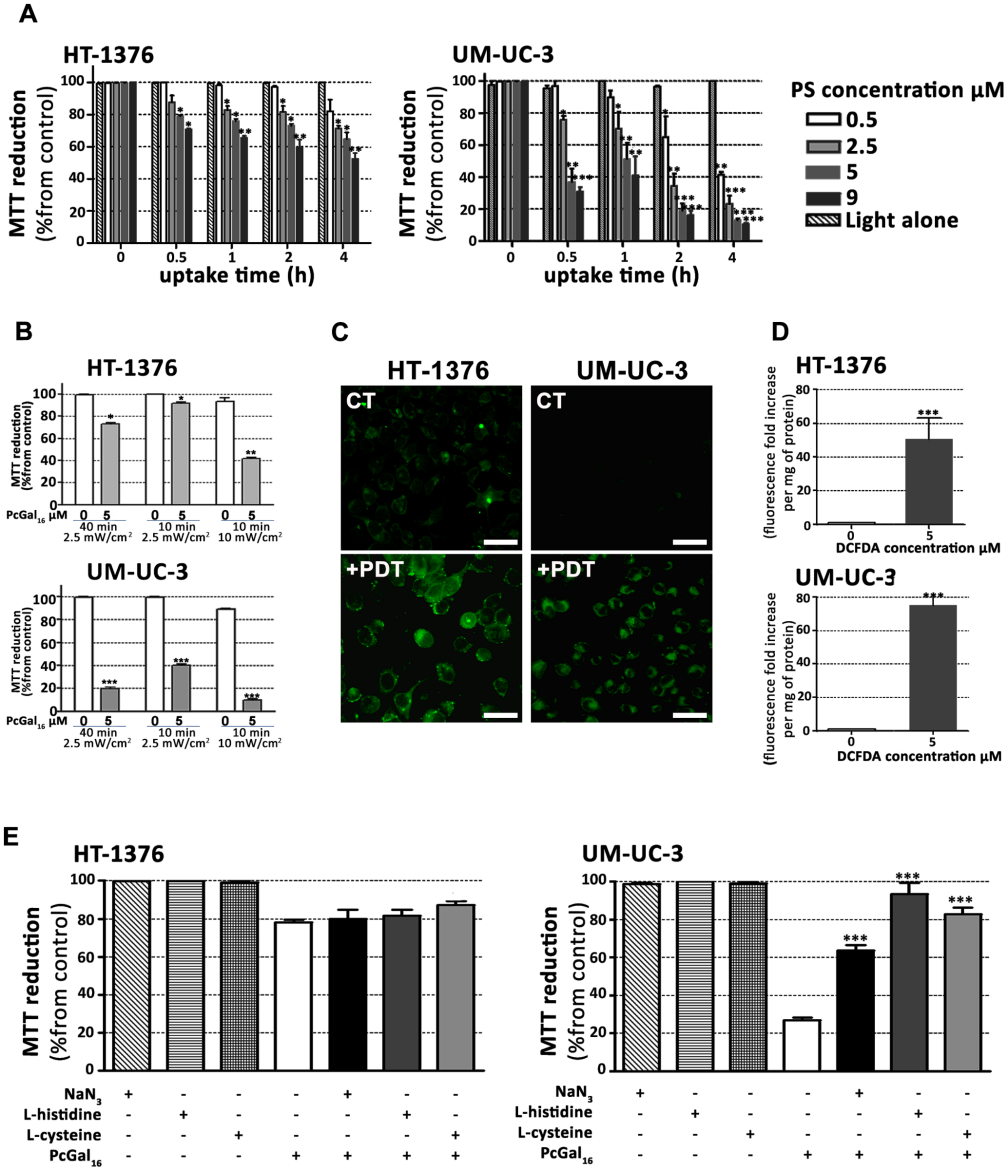
PcGal_16_ generates ROS and produces toxicity after PDT. Photocytotoxic effects after PcGal_16_-PDT in HT-1376 and UM-UC-3 cells evaluated 24 h after PDT using the MTT assay (panel A). The percentage of toxicity was calculated relatively to control cells (cells incubated with PBS and irradiated). Data are the mean ± S.D. of at least three independent experiments performed in triplicates. *(p<0.05), **(p<0.001), ***(p<0.0001) significantly different from control cells. Irradiation dose-dependent cell death in response to PDT with PcGal_16_ (panel B). Cytotoxicity was assessed 24 h after treatment using the MTT assay. The percentage of cytotoxicity was calculated relatively to control cells (untreated cells). Data are the mean value ± S.D. of at least three independent experiments performed in triplicates. *(p<0.05), **(p<0.001), ***(p<0.0001) significantly different from control cells. Representative fluorescence images (panel C) and quantification (panel D) of DCF fluorescence increase (as a measure of ROS production) in HT-1376 and UM-UC-3 cells, after PDT with PcGal_16_. *Scale bars* 20 µm. Data are the mean ± S.D. of at least three independent experiments performed in triplicates. *(p<0.05), ***(p<0.0001) significantly different from control cells Photocytotoxicity after PDT with PcGal_16_ in the presence of 50 nM of ROS quenchers (sodium azide, histidine and cysteine) in HT-1376 and UM-UC-3 cells (panel E). Cytotoxicity was assessed 24 h after treatment using the MTT assay. The percentage of cytotoxicity was calculated relatively to control cells (untreated cells). Data are the mean value ± S.D. of at least three independent experiments performed in triplicates. ***(p<0.0001) significantly different from MTT reduction (%) after PcGal_16_-PDT.

To test the effect of light irradiation (red light at 620–750 nm delivered at 2.5 mW/cm^2^ for 40 min, *i.e.* 6 J/cm^2^) after PcGal_16_ uptake on cell viability, MTT was performed 24 h after treatment (Figure 2). No cytotoxicity was observed in the untreated sham-irradiated cells (Figure 2A) or untreated irradiated cells in the presence of 0.45% (v/v) or less DMSO in PBS (data not shown). However, when HT-1376 and UM-UC-3 cells were incubated with PcGal_16_ and then irradiated, there was an increased phototoxicity in a concentration- and uptake time-dependent manner (Figure 2A). Data showed that PcGal_16_ exerted a higher phototoxicity on UM-UC-3 cells compared to HT-1376 cells (Figure 2A). Moreover, the percentage of cell death in treated cells compared to untreated cells was significantly influenced by the dose of light (Figure 2B). The phototoxicity was higher in cells irradiated at 6 J/cm^2^ than in cells irradiated at 1.5 J/cm^2^ (cells irradiated with light at 2.5 mW/cm^2^ for 40 min or 10 min, respectively). On the other hand, irradiation of cells with light at 10 mW/cm^2^ for 10 min (*i.e.* 6 J/cm^2^) resulted in induction of cell death in untreated control cells. In subsequent experiments, we then performed cells irradiation with light at 2.5 mW/cm^2^ for 40 min. Based on the uptake results (Figure 1A) and MTT data before (Figure S2) and after PcGal_16_ photoactivation (Figures 2A and 2B), we estimate the lowest concentration of PcGal_16_ and the lowest dose of light necessary to achieve high phototoxicity for both bladder cancer cell lines. When cells were incubated with 5 µM PcGal_16_ for 2 h and then irradiated with light at 6 J/cm^2^ (cells irradiated for 40 min with light at 2.5 mW/cm^2^), we observed a significant increase in phototoxicity of HT-1376 and UM-UC-3 cells. The cells were also incubated with 5 µM of PcF_16_ during 2 h and then irradiated. As shown in Figures S2 and 2, the phototoxicity was higher for PcGal_16_ than for non-conjugated PcF_16_. Based on the critical role of ROS in causing cell death after PDT and considering the different PDT-induced phototoxicity observed in UM-UC-3 and HT-1376 cells, the intracellular production of ROS was evaluated immediately after PDT in the cells previously incubated with 5 µM PcGal_16_ for 2 h. The application of PcGal_16_ in combination with PDT led to a high significant augmentation of ROS in both bladder cancer cell lines compared with the control (Figures 2C and 2D). The ROS levels (DCF fluorescence fold increase per mg of protein) in HT-1376 and UM-UC-3 cells were 50.52±12.77 and 74.88±11.49, respectively, when 5 µM H_2_DCFDA was used for ROS detection (Figure 2D).

To assess the contribution of ROS in PcGal_16_-mediated cell death, quenchers of ROS (histidine, sodium azide [38] and cysteine [39]) were added at non-toxic concentrations to the incubation medium when the cells were irradiated. Cell viability evaluated 24 h after treatment was dependent on the used scavenger and cell type (Figure 2E). For the cell line UM-UC-3, all quenchers at the employed concentration partially decrease the PcGal_16_–PDT-induced phototoxicity. For the cell line HT-1376, none of the quenchers used in these experiments were able to reduce the phototoxicity induced by photoactivated PcGal_16_.

**Figure 3:**
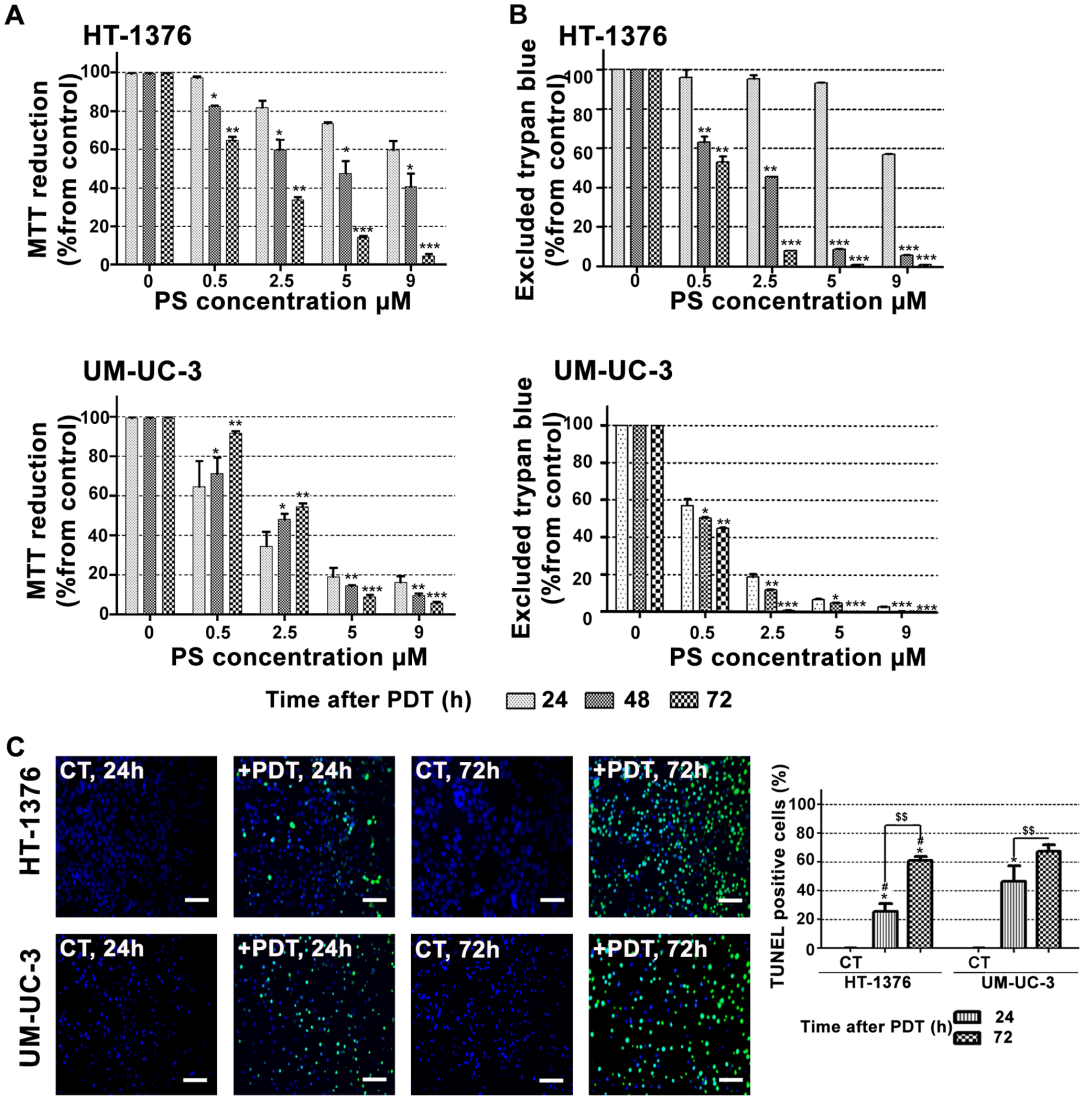
PDT with PcGal_16_ has a long-term phototoxicity effect. Cytotoxicity was assessed 24, 48, and 72_16_-PDT using the MTT (panel A) and trypan blue staining (panel B) assays. The percentage of cytotoxicity was calculated relatively to control cells (cells incubated with PBS in darkness and then irradiated) at the respective uptake time. Data are the mean value ± S.D. of at least three independent experiments performed in triplicates. *(p<0.05), **(p<0.001), ***(p<0.0001) significantly different from MTT reduction (%) or excluded trypan blue (%) at 24 h after PDT for the respective concentration. Representative fluorescence images revealing cell death in HT-1376 and UM-UC-3 cells after PDT with PcGal_16_ by TUNEL staining 24 and 72 h after treatment (panel C). DAPI was used for nuclei staining (blue) and TUNEL staining was used to visualize dead cells (green). *Scale bars* 20 µm. Quantification of TUNEL-positive cells 24 and 72 h after PDT with PcGal_16_. *(p<0.05) significantly. different from control cells. ^$$^(p<0.001) significantly different from TUNEL-positive cells 24 h after PDT. ^#^(p<0.05) significantly different from TUNEL-positive UM-UC-3 cells at the respective time after PDT.

To assess whether PDT has a long-term phototoxic effect, we evaluated cell viability for up to 72 h after PDT treatment. In both cell lines, the results obtained with the MTT colorimetric assay (cell metabolic activity) were correlated with the loss of cell membrane integrity (trypan blue staining) (Figures 3A and 3B). Overall, UM-UC-3 and HT-1376 bladder cancer cells were unable to recover from the PDT-induced damage effects 48 or 72 h after treatment, for PcGal_16_ concentrations above 5 µM. TUNEL data revealed that there is an induction of cell death in a time-dependent manner in the cells irradiated after incubation with PcGal_16_ (Figure 3C). Twenty-four hours after PDT with PcGal_16_, the percentage of TUNEL positive cells in UM-UC-3 cell line was 1.8 higher than that of the HT-1376 cells, but after 72 h there was almost the same percentage of TUNEL-positive cells in both cell lines. The concentrations of PcGal_16_ necessary to inhibit the metabolic activity of UM-UC-3 and HT-1376 bladder cancer cells in 50% can be estimated from Figure 3A. These values, named as “photocytotoxic concentrations” (IC_50_) are reported in Table 1. Data show that 24 h after PDT, IC_50_ value is lower for UM-UC-3 when compared with HT-1376 cells and similar for these cell lines 72 h after PDT.

**Table 1:** Values for photocytotoxic concentration (IC_50_, µM) of photoactivated PcGal_16_ on human bladder cancer cell lines, HT-1376 and UM-UC-3.

	HT-1376 cell line			UM-UC-3 cell line		
**Hours after PDT**	24	48	72	24	48	72
**IC_50_ (µM), CI_95%_**	-	3.3 [0.6;10.7]	2.5 [2.2;2.9]	2.1 [0.9;5.0]	2.8 [2.4; 3.2]	2.6 [2.6;2.7]

IC_50_ is the incubation concentration that inhibits the proliferation of cultures in 50%, after cells' incubation with **PcGal_16_** and irradiation. IC_50_ values were calculated using the MTT dose response curves (24, 48, and 72 h after PDT), obtained for cells incubated with **PcGal_16_** at various concentrations for 2 h.

CI_95%_: 95% Confidence interval.

### PcGal_16_ induces antioxidant enzyme response after photodynamic therapy

Considering the different levels of ROS produced in the two bladder cancer cell lines after PDT with PcGal_16_, we investigated (immediately after PDT) the involvement of specific antioxidant enzymes [40] in the detoxification of ROS and/or resulting toxic products. For that, the activities of the three major antioxidant enzymes, SOD, CAT, and GPox were determined by spectroscopy [41]. SOD catalyses the dismutation of superoxide radical anions into hydrogen peroxide and molecular oxygen. Hydrogen peroxide is then removed by CAT when it is present at high concentrations or by GPox when present at low concentrations. Knowing about the indirect antioxidant function [40] of GR in the replenishment of gluthathione levels in reduced form (GSH) and of GST in the elimination of reactive compounds through their conjugation with GSH, their activities were also determined.

In UM-UC-3 control cells, the activities of GR, SOD and CAT were 1.5-fold, 1.9-fold and 1.5-fold higher, respectively, than in HT-1376 control cells (Table 2). There was no significant difference in the activities of GST and GPox between the control cells of the two cell lines. After PDT with PcGal_16_, there was a 1.3-fold, 3.1-fold and 1.5-fold increase in the activities of GR, SOD and CAT in UM-UC-3 cells. In HT-1376 cells, there was a 2.2-fold, 4.6-fold and 4.8-fold increase in GR, SOD and CAT activities and a 2-fold decrease in the activity of GST after PDT with PcGal_16_. Treatment of HT-1376 resulted in a 2.3-fold increased of CAT activity as compared to UM-UC-3-treated cells. The ability of HT-1376 cells to produce an antioxidant adaptive response, activating the antioxidant enzymes GR, SOD and CAT can explain the higher resistance observed 24 h after PDT with PcGal_16_ as compared with UM-UC-3 cells.

**Table 2:** Values of activity (mU/mg of protein) of antioxidant enzymes superoxide dismutase (SOD), catalase (CAT), glutathione peroxidase (GPox), glutathione reductase (GR) and glutathione S-transferase (GST) determined after PDT.

Cell line	PcGal_16_-PDT	Enzyme activity (mU/mg of protein)				
		GST	GPox	GR	SOD	CAT
**UM-UC-3**	−	44.0±1.4	232.4±23.6	252.3±13.9$	56.0±13.1$	36.31±1.3$
	+	39.7±2.7	243.6±12.0	316.1±11.3#	173.6±4.8#	52.89±2.7#
**HT-1376**	−	50.4±2.4	236.1±9.9	163.4±1.9	29.56±1.9	24.85±2.2
	+	24.6±0.5$ ^,^ *	252.3±18.2	356.9±30.6$	134.7±4.3$ ^,^ *	119.8±3.3$ ^,^ *

$ (p<0.05): significantly different from HT-1376 control cells;

# (p<0.05): significantly different from UM-UC-3 control cells;

*(p<0.05): significantly different from UM-UC-3 treated cells.

### Knockdown of galectin-1 and GLUT1 decreases the uptake and phototoxicity of PcGal_16_

We investigated whether the presence of the dendritic galactose units around the core of Pc molecule could facilitate the interaction of this PS with specific domains in the plasma membrane of cancer cells. We hypothesized that domains enriched in carbohydrate-binding proteins [42] could facilitate the interaction with PcGal_16_, enhancing somehow its cellular uptake, and therefore its photodynamic potential.

**Figure 4:**
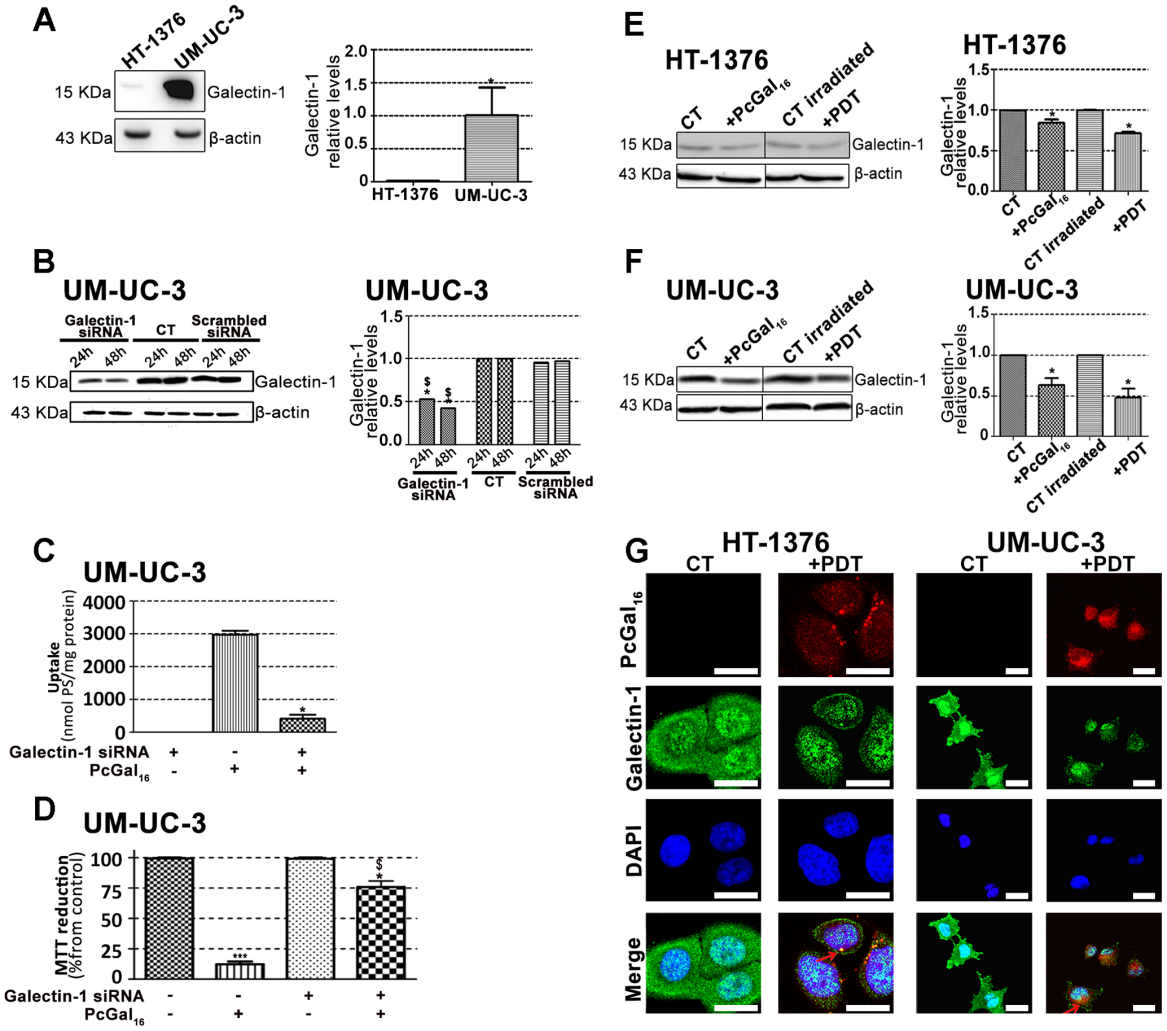
Knockdown of galectin-1 decreases de uptake and phototoxicity of PcGal_16_. Western blotting analysis and quantification of galectin-1 protein levels in HT-1376 and UM-UC-3 cells (panel A), in HT-1376 cells (panel E) or UM-UC-3 cells (panel F) after uptake with PcGal_16_ in darkness and after PDT. β-actin was blotted as loading control. Quantitative analysis of galectin-1 (normalized to β -actin) expressed as a ratio of the levels found in HT-1376 cells. *(p<0.05) significantly different from HT-1376 cells. Quantitative analysis of galectin-1 (normalized to β-actin) expressed as a ratio of the levels found in untreated HT-1376 or UM-UC-3 cells (panel E, F). Data represents mean ± S.D. of five independent experiments. *(p<0.05) significantly different from untreated HT-1376 or UM-UC-3 cells. Knockdown of galectin-1 in UM-UC-3 bladder cancer cells as determined by Western blotting 24 and 48 h post-transfection (panel B). Quantitative analysis of galectin-1 (normalized to β-actin) expressed as a ratio of the levels found in non-transfected control cells. Data represents mean ± S.D. of five independent experiments. *(p<0.05), ^$^(p<0.05) significantly different from non-transfected control cells or cells treated with scrambled siRNA, respectively. Intracellular uptake of PcGal_16_ by UM-UC-3 bladder cancer cells transfected with galectin-1 siRNA (panel C). The cells were incubated with PcGal_16_ 48 h post-transfection with galectin-1 siRNA. Data are the mean ± S.D. of at least three independent experiments performed in triplicates. *(p<0.05) significantly different from non-transfected control cells. Photocytotoxic effects after PcGal_16_-PDT in UM-UC-3 cells transfected with galectin-1 siRNA (panel D). Phototoxicity was evaluated 72 h after PDT. Data are the mean ± S.D. of at least three independent experiments performed in triplicates. *(p<0.05), ***(p<0.0001) significantly different from control cells. ^$^(p<0.05), significantly different from PDT with PcGal_16_ in non-transfected cells. Representative fluorescence images (panel G) of galectin-1 protein (green) in cancer cells before and after incubation with PcGal_16_ (red), with DAPI staining the nucleus (blue). *Scale bars* 20 µm.

**Figure 5:**
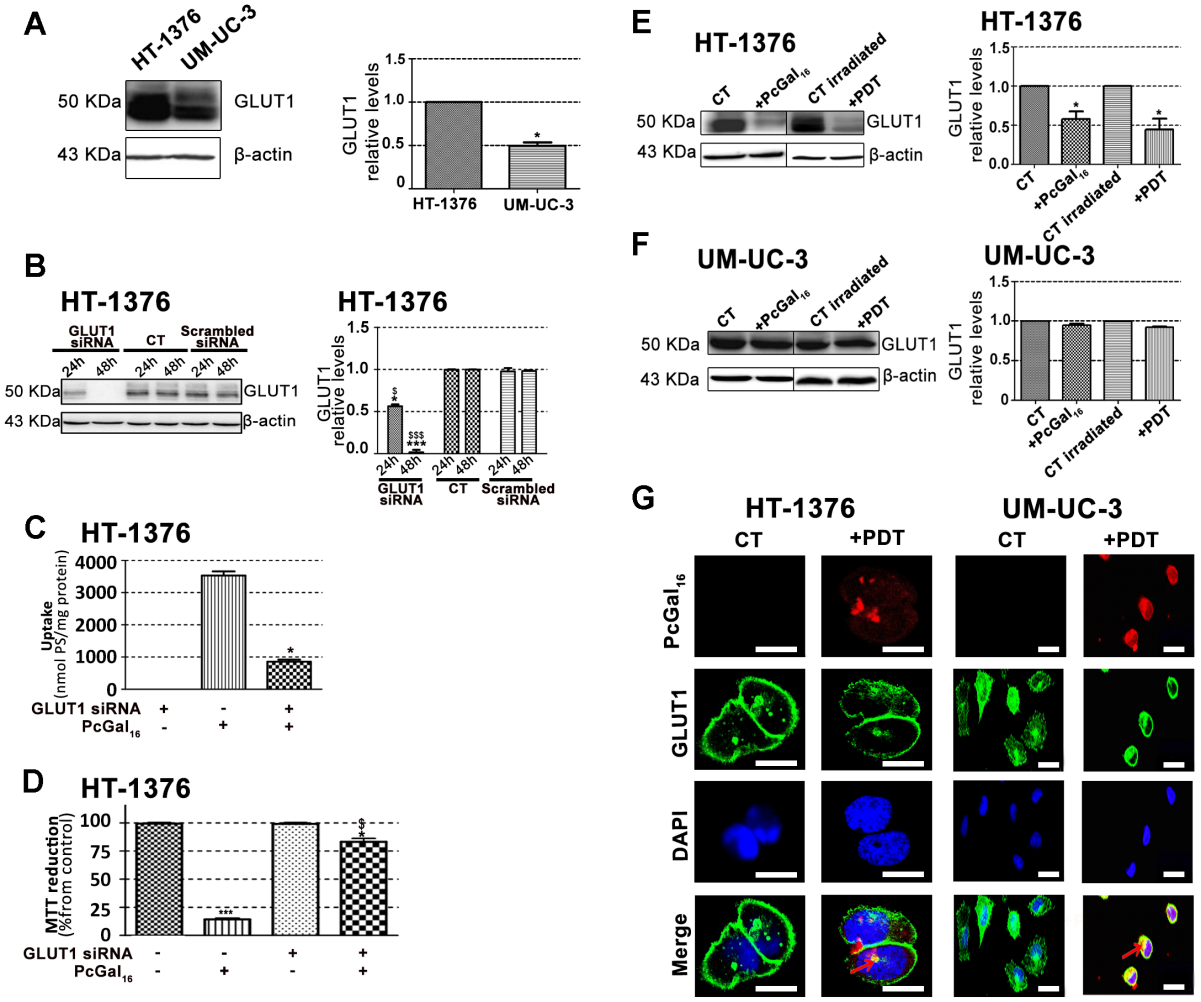
Knockdown of GLUT1 decreases de uptake and phototoxicity of PcGal_16_. Western blotting analysis and quantification of GLUT1 protein levels in HT-1376 and UM-UC-3 cells (panel A), in HT-1376 cells (panel E) or UM-UC-3 cells (panel F) after uptake with PcGal_16_ in darkness and after PDT. β-actin was blotted as loading control. Quantitative analysis of GLUT1 (normalized to β-actin) expressed as a ratio of the levels found in HT-1376 cells (panel A). *(p<0.05) significantly different from HT-1376 cells. Quantitative analysis of GLUT1 (normalized to β-actin) expressed as a ratio of the levels found in untreated HT-1376 or UM-UC-3 cells (panel E, F). Data represents mean ± S.D. of five independent experiments. *(p<0.05) significantly different from untreated HT-1376 cells. Knockdown of GLUT1 in HT-1376 bladder cancer cells as determined by Western blotting 24 and 48 h post-transfection (panel B). Quantitative analysis of GLUT1 (normalized to β-actin) expressed as a ratio of the levels found in non-transfected control cells. Data represents mean ± S.D. of five independent experiments. *(p<0.05), ***(p<0.0001) significantly different from non-transfected control cells. ^$^(p<0.05), ^$$$^(p<0.0001) significantly different from cells treated with scrambled siRNA. Intracellular uptake of PcGal_16_ by HT-1376 bladder cancer cells transfected with GLUT1 siRNA (panel C). The cells were incubated with PcGal_16_ 24 h post-transfection. Data are the mean ± S.D. of at least three independent experiments performed in triplicates. *(p<0.05) significantly different from non-transfected control cells. Photocytotoxic effects after PcGal_16_-PDT in UM-UC-3 cells transfected with galectin-1 siRNA (panel D). Phototoxicity was evaluated 72 h after PDT. Data are the mean ± S.D. of at least three independent experiments performed in triplicates. *(p<0.05), ***(p<0.0001) significantly different from control cells. *(p<0.05), significantly different from PDT with PcGal_16_ in non-transfected cells. Representative fluorescence images (panel G) of GLUT1 protein (green) in HT-1376 and UM-UC-3 cells before and after incubation with PcGal_16_ (red), with DAPI staining the nucleus (blue). *Scale bars* 20 µm.

Galectin [18] and glucose [19] proteins are expressed in high levels in cancer cells and both have affinity for galactose molecules. Therefore, we have evaluated the protein levels of galectin-1 and GLUT 1 in UM-UC-3 and HT-1376 cells, by Western Blotting and immunofluorescence (Figures 4 and 5).

The galectin-1 protein levels were higher in UM-UC-3 than in HT-1376 control cells (Figure 4A). To determine whether galectin-1 plays a role in the uptake of PcGal_16_ by cancer cells, siRNA was used to knockdown galectin-1 within UM-UC-3 bladder cancer cells. The treatment of UM-UC-3 cells with a pool of three target-specific siRNAs maximally suppressed galectin-1 by ≈50% at 24 h and 48 h post-transfection (Figure 4B), without affecting the expression of the housekeeping protein β-actin. The transfected cells were then treated with PcGal_16_ 48 h post-transfection. As shown in Figures 4C and 4D, transfected cells displayed a markedly decreased uptake and phototoxicity of PcGal_16_. The GLUT1 protein levels were higher in HT-1376 than in UM-UC-3 control cells (Figure 5A). Therefore, HT-1376 bladder cancer cells were also treated with a pool of three target-specific GLUT1 siRNAs. Application of GLUT1 siRNA suppressed GLUT1 by ≈50% and ≈90% at 24 h and 48 h post-transfection, respectively (Figure 5B). Treatment of HT-1376 cells with PcGal_16_ twenty-four hours post-transfection, resulted in a substantial decrease in the uptake and phototoxicity (Figures 5C and D).

### PcGal_16_ decreases the galectin-1 and GLUT1 protein levels

To further explore the role of galectin-1 and GLUT1 in the photodynamic effect induced by PcGal_16_, we determined the levels of these proteins before and after PDT. Both incubation of cancer cells with PcGal_16_ (*i.e.* incubation of cancer cells with PcGal_16_ in darkness) and PDT with PcGal_16_ induced a decrease in galectin-1 as observed by Western Blotting and immunofluorescence (Figures 4E, 4F and 4G). The decrease observed in galectin-1 was higher in UM-UC-3 cells as compared to HT-1376 cells and it was more evident after PDT. Using confocal fluorescence microscopy, we observed co-localization of PcGal_16_ with galectin-1 inside bladder cancer cells (Figure 4G).

Similar to what was observed for galectin-1, there was also a decrease in GLUT1 (Figures 5E, 5F and 5G) both after PcGal_16_ uptake and after PDT treatment in HT-1376 cancer cells. Furthermore, in these cancer cells it was higher after PDT than after PcGal_16_ uptake in darkness. In UM-UC-3 cells, PcGal_16_ was not able to reduce GLUT1 protein levels (Figure 5F). In both bladder cancer cell lines there was co-localization of PcGal_16_ with GLUT1 (Figure 5G). Overall, these findings clearly indicate show the critical involvement of the carbohydrate-binding proteins in the potential of PcGal_16_ as a therapeutic agent.

## Discussion

**Figure 6:**
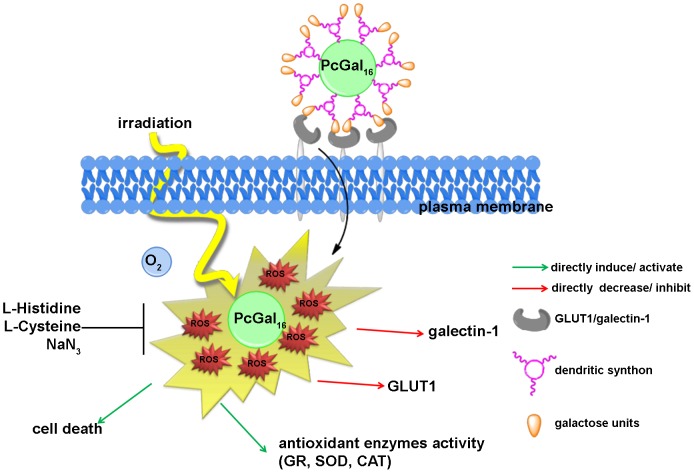
Hypothetic illustration of phototoxicity of PcGal_16_ in human bladder cancer cells. The uptake of PcGal_16_ by bladder cancer cells is modulated by the presence of carbohydrate-binding proteins present at the cell surface (*i.e.* GLUT1 and galectin-1). PcGal_16_ is a nontoxic compound *per se*, and has high photocytotoxic efficiency against bladder cancer cell lines. Treatment with ROS quenchers demonstrated that cell death in bladder cancer cells is mediated by the production of ROS after PDT. Immediately after PDT with PcGal_16_ there is an increase on the activity of antioxidant enzymes (SOD, CAT and GR antioxidant enzymes). The photoactivated PcGal_16_ co-localizes with galectin-1 and GLUT1and reduces their levels.

Third-generation PSs such as Pc coupled to carbohydrates are interesting for PDT, because they can be recognized by glycoprotein-based membrane proteins that are overexpressed in tumors [6]. Besides the enhancement of cellular recognition, the presence of dendritic galactose molecules improves Pc solubility and biocompatibility [34]. We have recently reported the synthesis of a new Pc with dendrimers of galactose sugar (PcGal_16_) that has valuable spectroscopic and photochemical properties [34]. In this study, we showed that PcGal_16_ is a nontoxic compound *per se*, and has high photocytotoxic efficiency in two bladder cancer cell lines, which is paralleled with its high ability to produce ROS and to induce oxidative stress (Figure 6).

The high intracellular uptake of the glycoconjugated PS, PcGal_16_, can be explained by the presence of carbohydrate cellular transporters or receptors present at the cell surface. Although the PcGal_16_ uptake was quite similar in the two bladder cancer cell lines, the expression of carbohydrate-binding proteins GLUT1 and galectin-1 is different amongst them. Besides its role in the import and export of glucose [19], the isoform of glucose transporter GLUT1 also transports D-galactose [19] having lower affinity for it than for D-glucose. Studies have been suggested that the hydroxyl groups in C1, C3 and C4 positions of D-galactose are hydrogen bond acceptors for GLUT1 sugar uptake site [43]. Like other galectins, galectin-1 has a carbohydrate-recognition domain (CRD) able to recognize and bind β-galactose [17]. Our assays demonstrated that galectin-1 and GLUT1 are both expressed by UM-UC-3 and HT-1376 cells. However, HT-1376 cells present higher GLUT1 levels compared with UM-UC-3 cells, and the contrary was observed for galectin-1. Although a similar PcGal_16_ uptake was observed in the two bladder cancer cell lines, both GLUT1 and galectin-1 may contribute for its specificity modulating the intracellular uptake. Knockdown of galectin-1 and GLUT1 in UM-UC-3 and HT-1376 cells, respectively, was associated with a marked decrease of PcGal_16_ uptake and phototoxicity. Together, these data demonstrated that galectin-1 and GLUT1 contribute for the efficacy of PDT mediated by PcGal_16_.

Interestingly, although the similar uptake of PcGal_16_ by UM-UC-3 and HT-1376 cells, the phototoxicity induced 24 h after PDT was higher in UM-UC-3 cells than in HT-1376 cells. Such lack of association between uptake and phototoxicity has been described [44], [45]. We investigated whether the higher phototoxicity observed in UM-UC-3 cells was due to higher production of ROS and/or higher oxidative damage compared with that in HT-1376 cells. As expected, the ability of PcGal_16_ to produce ROS was higher in UM-UC-3 than in HT-1376 cells.

In PDT, it has been described that ROS can be generated by two photochemical reactions [46], [47]. In type-II photochemical reactions, the excited PS in its triplet state can transfer its energy to molecular oxygen leading to the formation of singlet oxygen. Type-I photochemical reactions happen when an excited PS reacts with a biological substrate forming radicals and radical ions. Treatment with ROS quenchers demonstrated that in UM-UC-3 cells, singlet oxygen should have a high effect since cell death was highly reduced with quenchers of singlet oxygen (sodium azide and histidine). Further studies are needed to gain insight into the contribution of specific ROS in PcGal_16_-mediated cell death after PDT.

Interestingly, we observed that PDT with PcGal_16_ has a long-term phototoxic effect in both cancer cell lines. Cytotoxicity assays (MTT, trypan blue and TUNEL assays) performed 72 h after PDT demonstrated that UM-UC-3 cells were not able to recover. Moreover, in HT-1376 cells there was a marked induction of cell death occurring from 24 to 72 h after PDT with PcGal_16_. The three distinct cytotoxic methods used in the present work are widely applied in the study of cell death: MTT (indicator of metabolic activity), trypan blue staining (indicator of membrane integrity loss occurring in necrosis or in late stages of apoptosis) and TUNEL assay (indicator of DNA fragmentation, a key factor of apoptosis). Cell death in PDT may occur by apoptosis or necrosis, or even by a combination of the two mechanisms [2]. A more specific and comprehensive study is needed to understand the specific cell death pathways induced after PDT with PcGal_16_ in the bladder cancer cells used in this study. The different cell death obtained 24 h after PDT in UM-UC-3 and HT-1376 cells can be partially explained by the different amount of ROS present in both cells lines after irradiation. In addition, the resistance exhibited by HT-1376 cells could be due to the presence of efficient protective mechanisms, at least in the first stages after photodynamic treatment. Cytoprotective mechanisms initiated by cancer cells after PDT are well-known [47]. The increase of antioxidant molecules (*e.g.* gluthathione, vitamin C and vitamin E) [48] and the induction of genes encoding proteins involved in apoptosis or in the repair of lesions [49] are two of the well-known cytoprotective mechanisms induced after PDT. Another one is based on the equilibrium between photo-oxidative impairment of cells by ROS *versus* elimination of ROS by the activity of cellular antioxidant enzymes. Recent studies have shown that PDT caused increased-antioxidant enzymes activity and expression [50]. Thus, PDT efficacy can be influenced by the antioxidant response of the enzymes SOD, the GSH system and CAT.

Our data demonstrated that after PDT with PcGal_16_ there was an increase in the activity of SOD, CAT and GR antioxidant enzymes in both cell lines, being higher in HT-1376 than in UM-UC-3 cells. This higher antioxidant defense of HT-1376 cells against ROS can explain the results obtained 24 h after treatment. However, it is hypothesized that this was not maintained for 72 h after PDT since for this time point there was a massive cell death. This not only suggests that in this cell line there is a temporal relationship between ROS levels and cell death, but shows that antioxidant enzymes activity is of greater importance in protecting HT-1376 cells for at least 24 h after PDT with PcGal_16_. Regarding the activity of antioxidant enzymes, in HT-1376 cells it was also observed a decrease in the activity of GST, which is an enzyme implicated in cells defense against oxidation products. This enzyme has been described as protecting cells from DNA desintegration and drug toxicity [51]. GST isoforms are overexpressed in multidrug resistant tumors having an important role in tumors drug resistance by direct detoxification or inhibition of the MAP kinase pathway [51]. Thus, the higher cell death observed in HT-1376 cells 72 h after treatment can be also related with the activity of GST. A decrease in the activity of GST can be associated with DNA fragmentation and cell death 72 h after treatment.

Understanding the role of galactose moieties in the recognition of the PS by cancer cells may allow the investigation and development of more focused therapeutic strategies. Thus, we investigated whether PcGal_16_ could be directly recognized by specific carbohydrate-binding proteins present at the plasma membrane. Consistently, the photoactivated PcGal_16_ was shown to co-localize and reduce the levels of the plasma membrane proteins galectin-1 and GLUT1. Moreover, the immunofluorescence and Western Blotting studies demonstrated that, although its non-dark toxicity, PcGal_16_ decreases the levels of galectin-1 and GLUT1 proteins. A plausible explanation for the decreased levels of the galactose binding proteins, galectin-1 and GLUT1, after incubation with PcGal_16_ can be the masking of the epitope, which can block antibody-epitope binding due to changes in protein conformation or, eventually, endocytosis of these proteins and subsequent degradation. Thus, the changes observed in the levels of galectin-1 and GLUT1 could be induced directly by the binding of PcGal_16_ to the carbohydrate-binding proteins and/or indirectly by the generation of ROS after PDT with PcGal_16_.

Although significant progress has been made in research related with the role of galectins in cancer, the information underlying the molecular mechanisms that control the expression of these proteins in tumour cells is scarce. The interaction of PSs with galectins (namely galectin-1 and galectin-3) has been studied by spectroscopic studies [52] and molecular modeling analysis [6], [27]; however, they have not been validated by *in vitro* studies. As far as we know, there are no *in vitro* reports indicating whether PSs can modulate the expression of carbohydrate-binding proteins such as galectin-1 and GLUT1. Knowing that galectin-1 expression is correlated with cell metastatic potential [18], [53] and contributes to tumor progression and resistance after conventional cancer therapeutic modalities [18], the ability of PcGal_16_ to reduce the levels of galectin-1 after its uptake and/or photoactivation prompted us to envisage PcGal_16_ as a potential candidate for cancer treatment.

Knowing that the overexpression of GLUTs is involved in tumor glycolysis - one of the biochemical “hallmarks” of cancer - the efficiency of PcGal_16_ as an efficient anti-cancer PS is also evidenced by its ability to reduce GLUT1. GLUT1 is an attractive target to consider in the development of new PSs because it is lower expressed in normal-epithelial tissues or benign epithelial cell tumors when compared with human cancer cells [54]. The function of GLUT1 in the tumorogenesis process has been demonstrated by *in vitro* and *in vivo* studies, where the overexpression of GLUT1 antisense resulted in the inhibition of HL60 leukaemia cells proliferation and MKN-45 derived xenografs, respectively [55], [56]. Based on the results of the current study, we envisage PcGal_1**6**_ as a promising therapeutic agent for the treatment of bladder cancer. Further studies are warranted to investigate the selectivity and photototoxicity of this PS in an *in vivo* model of bladder cancer, to contribute to a possible impact on clinical practice.

## Supporting Information

Figure S1 Chemical structures of free phthalocyanine PcF_16_ and galacto-dendrimer phthalocyanine PcGal_16_.
(TIF)

Figure S2 PcGal_16_ is non-toxic in darkness, PcF_16_ is non-toxic in darkness and after PDT. Non-dark toxicity of various concentrations of PcGal_16_ in HT-1376 and UM-UC-3 cells (panel A). Non-dark toxicity was assessed using the MTT colorimetric assay 24, 48, and 72 h after treat HT-1376 and UM-UC-3 cells (panel B). Toxicity of PcF_16_ at 5 µM in darkness and after PDT (panel C) in HT-1376 and UM-UC-3 cells. The toxicity was assessed using the MTT colorimetric assay 24 h after treat HT-1376 and UM-UC-3 cells. Data are the mean ± S.D. of at least three independent experiments performed in triplicates.
(TIF)
