# Supplementary figures and images for: Galactodendritic Phthalocyanine Targets Carbohydrate-Binding Proteins Enhancing Photodynamic Therapy
Source: PLoS One. 2014 Apr 24;9(4):e95529. doi: 10.1371/journal.pone.0095529 (PMC3999036; doi:10.1371/journal.pone.0095529)

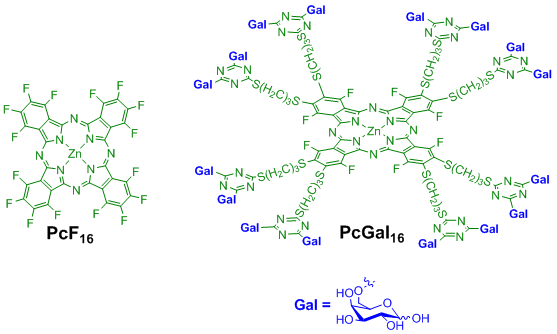

Supplement: Figure S1 — Chemical structures of free phthalocyanine PcF16 and galacto-dendrimer phthalocyanine PcGal16. (TIF) [file pone.0095529.s001.tif]

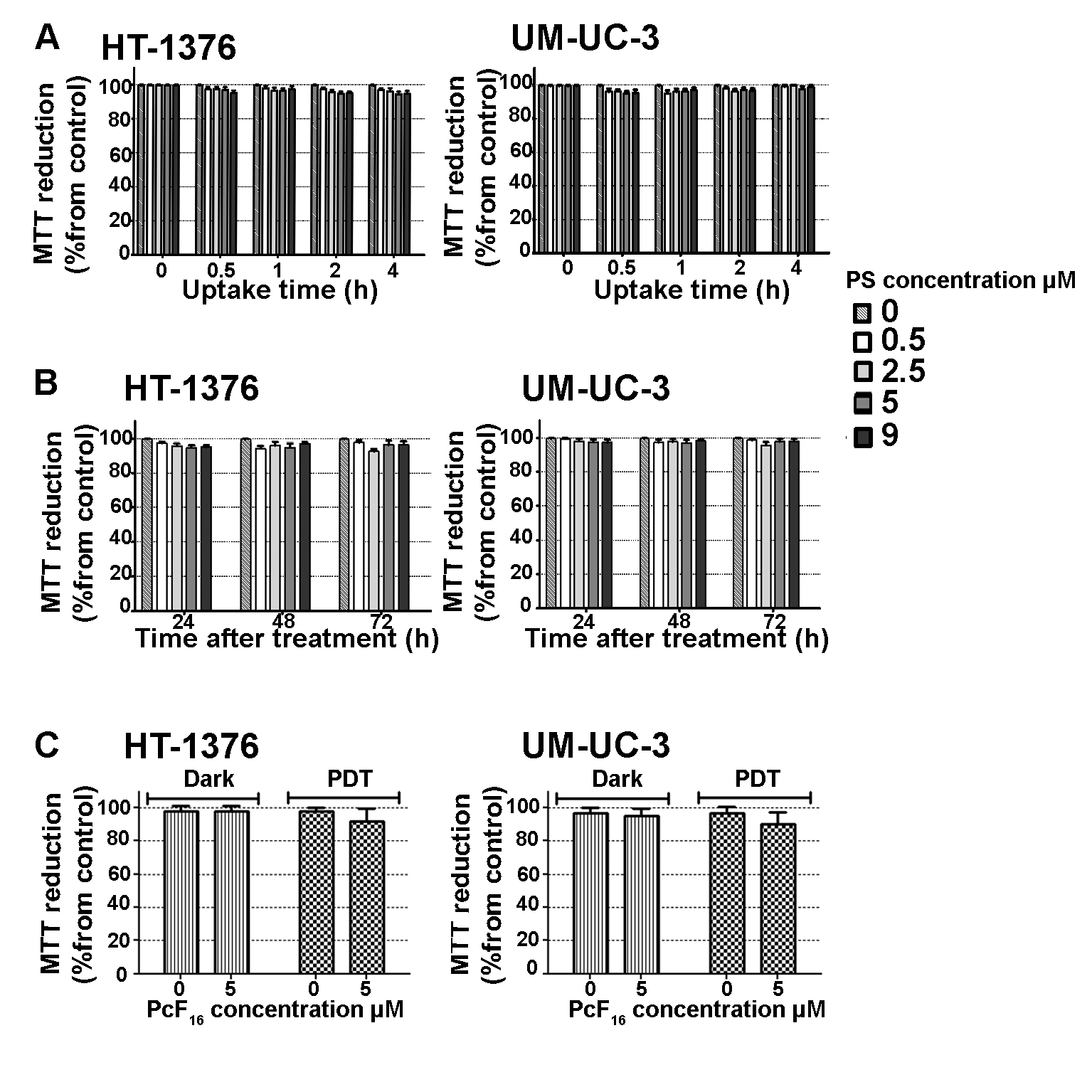

Supplement: Figure S2 — PcGal16 is non-toxic in darkness, PcF16 is non-toxic in darkness and after PDT. Non-dark toxicity of various concentrations of PcGal16 in HT-1376 and UM-UC-3 cells (panel A). Non-dark toxicity was assessed using the MTT colorimetric assay 24, 48, and 72 h after treat HT-1376 and UM-UC-3 cells (panel B). Toxicity of PcF16 at 5 µM in darkness and after PDT (panel C) in HT-1376 and UM-UC-3 cells. The toxicity was assessed using the MTT colorimetric assay 24 h after treat HT-1376 and UM-UC-3 cells. Data are the mean ± S.D. of at least three independent experiments performed in triplicates. (TIF) [file pone.0095529.s002.tif]
